# Genotyping and Molecular Characterization of Infectious Bursal Disease Virus Identified in Important Poultry-Raising Areas of China During 2019 and 2020

**DOI:** 10.3389/fvets.2021.759861

**Published:** 2021-12-01

**Authors:** Nan Jiang, Yulong Wang, Wenying Zhang, Xinxin Niu, Mengmeng Huang, Yulong Gao, Aijing Liu, Li Gao, Kai Li, Qing Pan, Changjun Liu, Yanping Zhang, Hongyu Cui, Xiaomei Wang, Xiaole Qi

**Affiliations:** ^1^Avian Immunosuppressive Diseases Division, State Key Laboratory of Veterinary Biotechnology, Harbin Veterinary Research Institute, The Chinese Academy of Agricultural Sciences, Harbin, China; ^2^Jiangsu Co-innovation Centre for Prevention and Control of Important Animal Infectious Disease and Zoonosis, Yangzhou University, Yangzhou, China

**Keywords:** infectious bursal disease virus, genotype, A2dB1, A3B3, molecular characterization

## Abstract

Infectious bursal disease (IBD) is an acute and highly contagious immunosuppressive disease caused by the infectious bursal disease virus (IBDV), which seriously threatens the healthy development of the poultry industry. Since its spread to China in the early 1990s, the very virulent IBDV (vvIBDV) characterized by high lethality, has been the focus of prevention and control. However, the novel variant IBDV (nVarIBDV), which has been widely prevalent in China since 2017, has brought a new threat to the poultry industry. In this study, the prevalence of IBDV in the important poultry-raising areas of China from 2019 to 2020 was detected. Of these, 45.1% (101/224) of the samples and 61.9% (26/42) of the chicken flocks were shown to be positive for IBDV. For 50 IBDVs, the sequences of the hypervariable region of the VP2 gene in segment A and of the B-marker of the VP1 gene in segment B were analyzed. The results revealed the coexistence of a number of different IBDV genotypes, including A2dB1 (nVar, 26/50, 52.0%), A3B3 (HLJ0504-like, 15/50, 30.0%), A1B1 (classical, 1/50, 2.0%), and A8B1 (attenuated, 1/50, 2.0%). This indicated that the newly emerging nVarIBDV of A2dB1 and the persistently circulating HLJ0504-like vvIBDV of A3B3 are the two important epidemic strains. Furthermore, we established that segment reassortment has occurred among these circulating strains. This study is the first to reveal the novel epidemic characteristics of IBDV since the report of the emerging nVarIBDV of A2dB1 in China.

## Introduction

Infectious bursal disease (IBD), also known as the Gumboro disease, is an economically important viral disease that affects young birds and causes acute death or immunosuppression (1). It is characterized by bursal damage secondary to an invasion of immature lymphocytes. The causative agent is the IBD virus (IBDV), which is a typical non-enveloped, icosahedral, double-stranded RNA virus belonging to the genus *Avibirnavirus* of the *Birnaviridea* family (2, 3). The genome of IBDV comprises segments A and B. Segment A contains two partially overlapping open reading frames (ORFs): the smaller ORF encodes the VP5 protein, while the large ORF encodes a polyprotein (NH_2_-pVP2-VP4-VP3-COOH) (4). VP2 is the viral capsid protein and the main protective antigen of IBDV. The hypervariable region (HVR) of VP2 (amino acids 206–350) is a fragment representative of the gene characteristics of segment A, which is widely used in analyses of the genetic evolution of IBDV (3, 5). Segment B encodes only a VP1 protein with RNA-dependent RNA polymerase (RdRp) activity. Similar to the HVR of VP2, the B-marker of VP1 (amino acids 110–252) is a fragment representative of the gene characteristics of segment B (6).

The IBDV was first reported in Gumboro (USA) in 1957, and is now defined as the classical IBDV (cIBDV). However, subsequent to its discovery, IBDV also underwent two large-scale mutational events (7). During the early 1980s, antigenic variant strains of IBDVs (varIBDVs) emerged in the USA, which were not fully controlled by the existing antibodies to cIBDV (8). Thereafter, during the late 1980s, a very virulent IBDV (vvIBDV), characterized by high lethality, broke out in Europe and subsequently spread worldwide (9), causing substantial economic losses to the global poultry industry. Nevertheless, with the widespread use of vaccines and improvements in poultry feeding and management, IBDV was gradually brought under effective control. However, since 2017, an atypical IBD caused by a novel variant of IBDV (nVarIBDV) has become widespread in China, evading the immuno-protection induced by the available vaccines against vvIBDV (10, 11). The lack of an effective antigen-matched vaccine has accordingly contributed to the continued prevalence of nVarIBDV. Recently, nVarIBDV epidemics have also been reported in Japan (12), South Korea (13), and Malaysia (14).

This study, conducted during 2019 and 2020, is part of the on-going efforts for monitoring the prevalence of IBDV in some of the main poultry-raising regions of China. The current epidemic characteristics were assessed on the basis of analyses of representative fragment sequences of viral genome segments A and B. This, to the best of our knowledge, is the first study to perform genotyping and molecular characterization of IBDV prevalent in the major poultry-raising regions of China since the emergence of nVarIBDV. We believe that our findings will contribute to the comprehensive prevention of IBD.

## Materials and Methods

### Samples

From 2019 to 2020, bursa samples were collected from poultry farms located in the main poultry-raising regions of China, on which birds (including commercial broilers, layers, and local breeds) were exhibiting suspected clinical symptoms of IBD. The research covered eight provinces, namely Heilongjiang, Liaoning, Shandong, Henan, Jiangsu, Hubei, Fujian, and Guangdong. All flocks on these farms had been vaccinated with commercially available IBD vaccines. The affected chickens, with ages ranging from 10 to 45 days, exhibited signs of weight loss and malaise, and some had become noticeably emaciated and had died subsequently. The collected bursa samples were cut into small pieces under aseptic conditions and transferred to sterile tubes containing steel beads, to which 1 mL phosphate-buffered saline (pH 7.2) was added; the mixture was grinded to produce a homogenate. These preparations were freeze–thawed thrice and centrifuged at 3,000 × g for 10 min at 4°C. The resulting supernatants were collected and stored at −80°C for subsequent detection.

### Viral RNA Extraction and Reverse Transcription Polymerase Chain Reaction

Total RNA was extracted from 200-μL aliquots of each bursal homogenate supernatant using RNAiso Plus (Takara Biotechnology Co., Ltd., Dalian, China) according to the manufacturer's instructions. The extracted RNA was reverse transcribed into cDNA with M-MLV reverse transcriptase (Invitrogen, Carlsbad, CA, USA). The cDNA was then used as a template to amplify IBDV gene fragments from segments A and B. For segment A, the primer pair P1 (5′-TCACCGTCCTCAGCTTAC-3′) and P2 (5′-TCAGGATTTGGGATCAGC-3′) was used to amplify a 643-bp fragment covering the HVR of the VP2 gene. For segment B, previously described (6) primers (B293U: 5′-TTTTGCAGCCGCGGTCTCT-3′ and B1008L: 5′-GTTTGACCCCTTTGTCCCTGC-3′) were used to amplify a 716-bp fragment covering the B-marker in the VP1 gene. A polymerase chain reaction (PCR) was performed using the following sequence: (1) initial denaturation at 95°C for 5 min, (2) 35 cycles at 95°C for 30 s, 56°C for 30 s, and 72°C for 1 min, and (3) final elongation at 72°C for 10 min. The PCR products were detected using 1% agarose gel electrophoresis. The positive reverse transcription PCR (RT-PCR) products were sequenced commercially by the Comate Biosciences Company (Changchun, China). The resulting nucleotide sequences of the HVR of the VP2 gene and the B-marker of the VP1 gene have been deposited in the GenBank database.

### Sequence Analysis

The nucleotide sequences of the VP2 HVR (Supplementary Table 1) and the VP1 B-marker (Supplementary Table 2) were aligned with those of reference strains obtained from GenBank using the Clustal X Software (version 2.0) (15). Maximum-likelihood (ML) trees were constructed using the MEGA6 program (16) based on the Kimura 2-parameter method with 1,000 replicates (17). The ML trees were generated using Interactive Tree Of Life (iTOL) (18).

## Results

### Detection of IBDV

A total of 224 bursa samples were collected from birds with suspected IBD from 42 chicken flocks on poultry farms located in eight main poultry-raising provinces in China between 2019 and 2020. These samples were subjected to an RT-PCR detection, which revealed that 45.1% (101/224) of the samples and 61.9% (26/42) of the flocks were positive for IBDV. Sequences of a 643-bp fragment of the VP2 gene (bp 491–1,133, corresponding to amino acids 165–377 covering the HVR [amino acids 206–350]) and of a 716-bp fragment of the VP1 gene (bp 182–897, corresponding to amino acids 61–299 covering the B-marker [amino acids 110–252]) of 50 representative IBDV strains from positive samples were determined and submitted to GenBank, the accession numbers of which are presented in Table 1.

**Table 1:** The IBDV strains identified in this study.

**No**.	**Strains**	**Phenotype^a^**	**Genotype**	**Source**	**Breed**	**Age (d)**	**Sampling date**	**GenBank accession no**.	
								**VP1**	**VP2**
1	IBDV-HLJ19-6102	VV	A3B3	Heilongjiang	Broiler	32	201906	MW863628	MW682898
2	IBDV-HLJ19-6101	VV	A3B3	Heilongjiang	Broiler	32	201906	MW863627	MW682897
3	IBDV-HLJ19-6003	VV	A3B3	Heilongjiang	Broiler	28	201906	MW863626	MW682896
4	IBDV-HLJ19-6002	VV	A3B3	Heilongjiang	Broiler	28	201906	MW863625	MW682895
5	IBDV-HLJ19-6001	VV	A3B3	Heilongjiang	Broiler	28	201906	MW863624	MW682894
6	IBDV-JS19-7105	nVar	A2dB1	Jiangsu	Local	35	201906	MW863636	MW682904
7	IBDV-JS19-7104	nVar	A2dB1	Jiangsu	Local	35	201906	MW863635	MW682903
8	IBDV-JS19-7103	nVar	A2dB1	Jiangsu	Local	35	201906	MW863634	MW682902
9	IBDV-JS19-7102	nVar	A2dB1	Jiangsu	Local	35	201906	MW863633	MW682901
10	IBDV-JS19-7101	nVar	A2dB1	Jiangsu	Local	35	201906	MW863632	MW682900
11	IBDV-SD19-9904	nVar	A2dB1	Shandong	Broiler	28	201907	MW863646	MT087549
12	IBDV-SD19-9903	nVar	A2dB1	Shandong	Broiler	28	201907	MW863645	MT087548
13	IBDV-SD19-9901	nVar	A2dB1	Shandong	Broiler	28	201907	MW863644	MT087547
14	IBDV-SD19-9804		A2dB3	Shandong	Broiler	28	201907	MW863643	MW682908
15	IBDV-SD19-9801		A2dB3	Shandong	Broiler	28	201907	MW863642	MW682907
16	IBDV-HLJ19-10903	AT	A8B1	Heilongjiang	Layer	28	201908	MW863629	MW682899
17	IBDV-JS19-13203	nVar	A2dB1	Jiangsu	Local	35	201909	MW863638	MT087551
18	IBDV-JS19-13202	nVar	A2dB1	Jiangsu	Local	35	201909	MW863637	MT087550
19	IBDV-JS19-13902	nVar	A2dB1	Jiangsu	Local	36	201910	MW863640	MT087553
20	IBDV-JS19-13204	nVar	A2dB1	Jiangsu	Local	35	201910	MW863639	MT087552
21	IBDV-HB19-15201	nVar	A2dB1	Hubei	Broiler	27	201911	MW863623	MW682893
22	IBDV-HB19-15102	nVar	A2dB1	Hubei	Broiler	27	201911	MW863622	MW682892
23	IBDV-GD19-15010		A2dB3	Guangdong	Broiler	10	201911	MW863621	MW682891
24	IBDV-GD19-15005	C	A1B1	Guangdong	Broiler	10	201911	MW863620	MW682890
25	IBDV-JS19-14601		A2dB3	Jiangsu	Local	28	201911	MW863641	MW682905
26	IBDV-SD20-9102		A3B1	Shandong	Broiler	35	202001	MZ766406	MZ766381
27	IBDV-SD20-9103		A3B1	Shandong	Broiler	35	202001	MZ766407	MZ766382
28	IBDV-SD20-9104		A3B1	Shandong	Broiler	35	202001	MZ766408	MZ766383
29	IBDV-HB20-4401	VV	A3B3	Hubei	Layer	45	202006	MZ766409	MZ766384
30	IBDV-HB20-4402	VV	A3B3	Hubei	Layer	45	202006	MZ766410	MZ766385
31	IBDV-HB20-4403	VV	A3B3	Hubei	Layer	45	202006	MZ766411	MZ766386
32	IBDV-HB20-4404	VV	A3B3	Hubei	Layer	45	202006	MZ766412	MZ766387
33	IBDV-HB20-4405	VV	A3B3	Hubei	Layer	45	202006	MZ766413	MZ766388
34	IBDV-HB20-4406	VV	A3B3	Hubei	Layer	45	202006	MZ766414	MZ766389
35	IBDV-HB20-4407	VV	A3B3	Hubei	Layer	45	202006	MZ766415	MZ766390
36	IBDV-HB20-4408	VV	A3B3	Hubei	Layer	45	202006	MZ766416	MZ766391
37	IBDV-HB20-4409	VV	A3B3	Hubei	Layer	45	202006	MZ766417	MZ766392
38	IBDV-HB20-4410	VV	A3B3	Hubei	Layer	45	202006	MZ766418	MZ766393
39	IBDV-SD20-6401	nVar	A2dB1	Shandong	Broiler	33	202007	MZ766419	MZ766394
40	IBDV-SD20-6402	nVar	A2dB1	Shandong	Broiler	33	202007	MZ766420	MZ766395
41	IBDV-SD20-6404	nVar	A2dB1	Shandong	Broiler	33	202007	MZ766421	MZ766396
42	IBDV-SD20-6406	nVar	A2dB1	Shandong	Broiler	33	202007	MZ766422	MZ766397
43	IBDV-SD20-6408	nVar	A2dB1	Shandong	Broiler	33	202007	MZ766423	MZ766398
44	IBDV-LN20-6605	nVar	A2dB1	Liaoning	Layer	25	202007	MZ766424	MZ766399
45	IBDV-FJ20-9401	nVar	A2dB1	Fujian	Broiler	30	202009	MZ766425	MZ766400
46	IBDV-FJ20-9402	nVar	A2dB1	Fujian	Broiler	30	202009	MZ766426	MZ766401
47	IBDV-FJ20-9404	nVar	A2dB1	Fujian	Broiler	30	202009	MZ766427	MZ766402
48	IBDV-FJ20-9405	nVar	A2dB1	Fujian	Broiler	30	202009	MZ766428	MZ766403
49	IBDV-FJ20-9406	nVar	A2dB1	Fujian	Broiler	30	202009	MZ766429	MZ766404
50	IBDV-FJ20-9407	nVar	A2dB1	Fujian	Broiler	30	202009	MZ766430	MZ766405

a *VV, very virulent strain; nVar, novel variant strain; AT, attenuated strain; C, classical strain*.

### Phylogenetic Analysis

**Figure 1:**
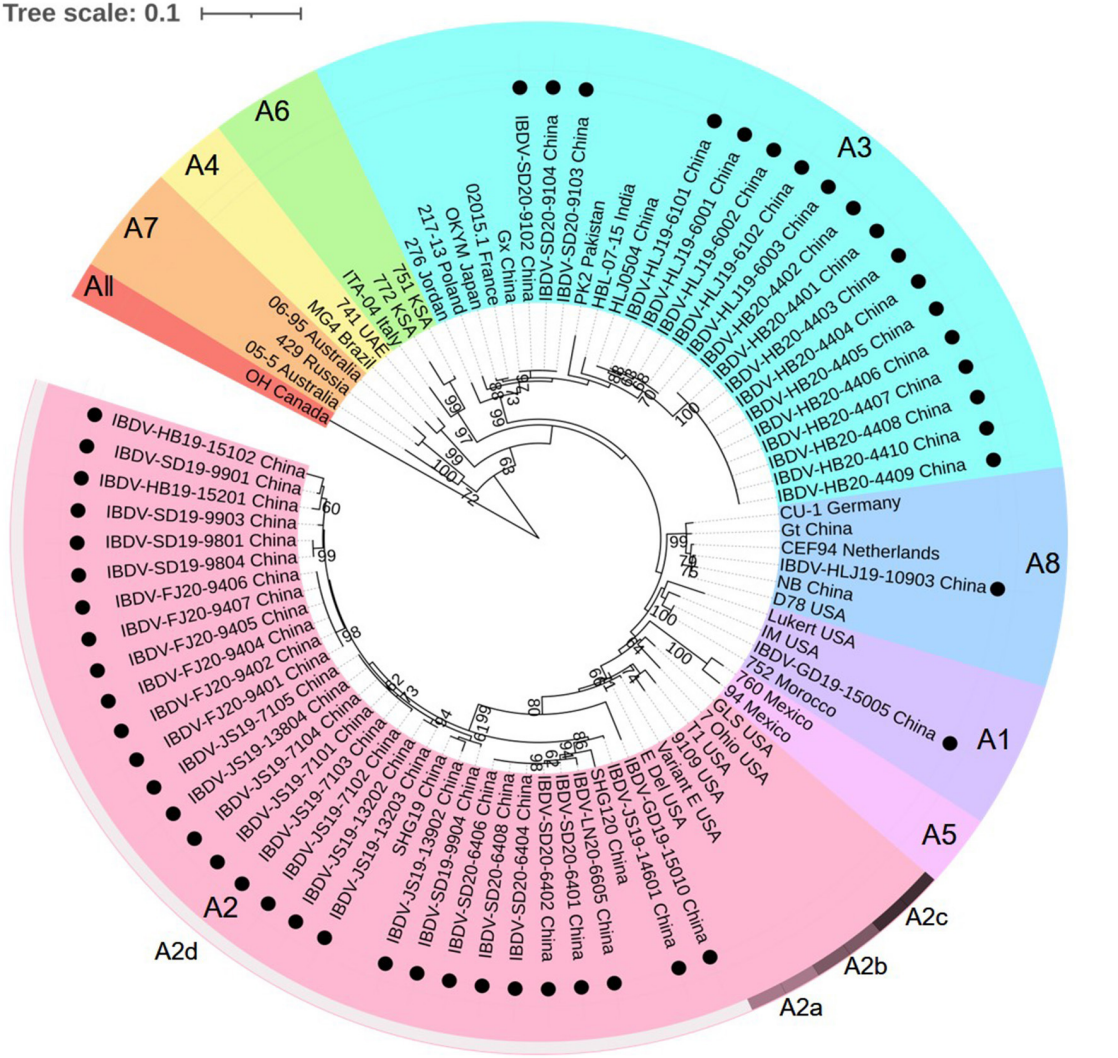
Phylogenetic analysis of the nucleotide sequences of the hypervariable region (HVR) of the VP2 gene. The tree was generated by the Maximum-likelihood method using MEGA6 Software. The tree was visualized in iTOL. The tree was drawn to scale, with branch lengths measured in the number of substitutions per site. Only branches supported by a bootstrap value above 60% were displayed. The analysis involved 35 reference nucleotide sequences, and 50 strains detected in this study were highlighted with a solid circle (•). Nine genogroups based on HVR of the VP2 gene in segment A were marked.

According to the current scheme used for IBDV genotype classification (19), genogroup is identified by individual segment and genotype is synthetically determined by both segments. A phylogenetic tree based on the nucleotide sequences of the VP2 HVR from reference strains and the strains identified in this study showed that serotype I strains were classified into eight genogroups (A1, A2, A3, A4, A5, A6, A7, and A8), whereas serotype II strains belonged to a single genogroup (Figure 1). Furthermore, genogroup A2 could be further divided into four lineages, namely A2a, A2b, A2c, and A2d. Among the 50 IBDV strains identified in this study, 30 (including the IBDV-HB19-15102 strain) were clustered into genogroup A2d of the novel variant IBDVs. Our analysis of the nucleotide sequence homology of the VP2 HVR revealed that these 30 IBDV strains showed high homology with the A2 genogroup IBDVs (89.1–99.5%), and particularly with the A2d genogroup strains (95.2–99.5%); these values were higher than those noted for the other genogroup strains (83.2–92.0%). A further 18 IBDV strains (including IBDV-HLJ19-6101) were clustered into the A3 genogroup of vvIBDVs, the homology with which was the highest (99.3–100%), whereas the homology with other strains was relatively lower (84.6–94.3%). One strain (IBDV-GD19-15005) was clustered with the classical IBDVs in genogroup A1 and exhibited a 95–100% similarity with the classical strains; this was higher than the similarity noted with the other strains (87.5–95.9%). Another single strain, IBDV-HLJ19-10903, was clustered in genogroup A8 containing attenuated strains, with which it showed a 98.2–99.5% similarity; its similarity to other genogroup strains was comparatively lower (86.1–96.1%).

**Figure 2:**
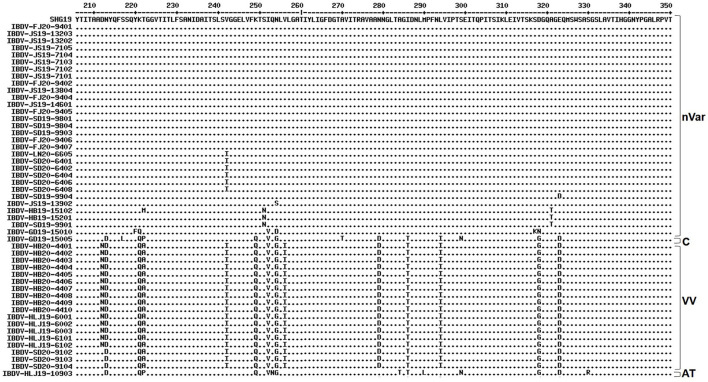
Amino acid substitution in the VP2 hypervariable region of the 50 strains detected in this study in comparison with the novel variant reference strain SHG19 (GenBank no. MH879092). Identical residues in aligned sequences were indicated by dots and differences were indicated by single letters. nVar, novel variant strain; C, classical strain; VV, very virulent strain; AT, attenuated strain.

Alignment of the characteristic amino acids of the HVR in VP2 (Figure 2) revealed that the 30 strains in genogroup A2d contained amino acids 222T, 249K, 286I, and 318D (characteristic of the varIBDVs) and also contained amino acids 221K, 252I, and 299S (unique to the nVarIBDVs). Similarly, the 18 strains clustering in genogroup A3 were found to contain amino acids 222A, 242I, 253Q, 256I, 279D, 284A, 294I, and 299S, which are typical of vvIBDV. The IBDV-HLJ19-10903 strain of genogroup A8 had characteristic amino acids including 222P, 279N, 284T, and 330R, which are consistent with those of attenuated strains. Conversely, the IBDV-GD19-15005 strain of genogroup A1 had amino acids 222P, 249Q, and 286T, which are characteristic of the classical strains.

**Figure 3:**
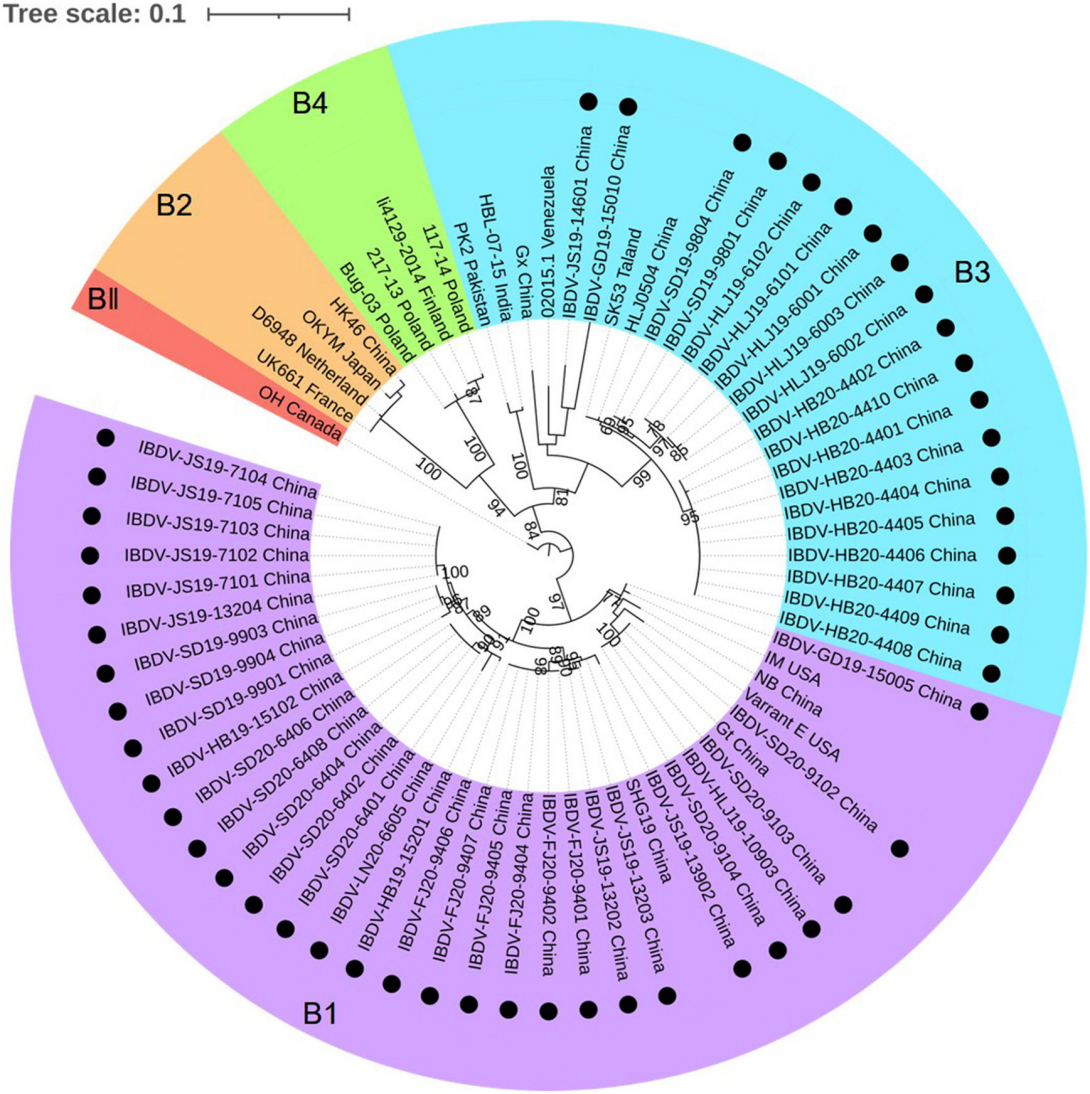
Phylogenetic analysis of the nucleotide sequences of the B-marker of the VP1 gene. The tree was generated by the maximum likelihood method using MEGA6 Software. The tree was visualized in iTOL. The tree was drawn to scale, with branch lengths measured in the number of substitutions per site. Only branches supported by a bootstrap value above 60% were displayed. The analysis involved 20 nucleotide sequences, and 50 strains detected in this study were highlighted with a solid circle (•). Five genogroups based on B-marker of the VP1 gene in segment B were marked.

The phylogenetic tree generated for the B-marker of the VP1 gene of the aforementioned 50 IBDV strains separated the serotype I strains into four clades: B1, B2, B3, and B4 (Figure 3), with strains carrying the B-marker clustering into the B1 and B2 clades. There were 31 IBDV strains clustering with genogroup B1; their nucleotide sequences showed a high homology with that of the representative genogroup strains (99.3–100%) and a lower homology with that of the strains in other genogroups (85.3–90.0%). The other 19 IBDV strains were found to cluster within the B3 genogroup of the HLJ0504-like strains and showed 89.1–99.1% similarity with these strains, which was higher than the 84.7–90.0% similarity noted with strains of the other genogroups.

Combining the characteristics of the representative regions of segments A and B, the 50 strains detected in this study could be divided into the following six genotypes: A2dB1 (26/50, 52%), A3B3 (15/50, 30%), A2dB3 (4/50, 8%), A3B1(3/50, 6%), A1B1 (1/50, 2%), and A8B1 (1/50, 2%). Among these, genotypes A2dB1, A3B3, A1B1, and A8B1 corresponded to the traditional phenotypes nVarIBDV, HLJ0504-like vvIBDV, classical IBDV, and attenuated IBDV, respectively. Moreover, genotypes A2dB3 and A3B1 are reassortment strains, the former containing segment A of nVarIBDV and segment B of HLJ0504-like vvIBDV, and the latter containing segment A from HLJ0504-like vvIBDV and segment B from the attenuated IBDV.

## Discussion

During the late 1980s, there was a sudden outbreak of the highly lethal vvIBDV in Europe, which spread rapidly to the other parts of the world and posed a substantial threat to the healthy development of the global poultry industry (9). Since the first reported incidence of vvIBDV in China during the early 1990s, the virus has caused serious economic losses to the national poultry industry. Nevertheless, concerted efforts for nearly 30 years, mainly including widespread vaccination and improved poultry feeding management, have succeeded in effectively bringing the vvIBDV epidemic under control. However, since 2017, nVarIBDV has become widely prevalent in the immunized chicken flocks in China, causing acute lesions of the bursa (the central immune organ of chickens) and leading to significant immunosuppression and pronounced weight reduction among the infected birds (10). This has further impeded immunization effects achieved using the avian influenza and Newcastle disease vaccines (11, 20).

As part of the efforts designed to keep track of the IBDV status, we continued to monitor the prevalence of IBDV in parts of Northeastern, Central, Southern, and Eastern China from 2019 to 2020. We accordingly established that the detected IBDV strains can be divided into the following six genotypes: A2dB1 (26/50, 52%), A3B3 (15/50, 30%), A2dB3 (4/50, 8%), A3B1(3/50, 6%), A1B1 (1/50, 2%), and A8B1 (1/50, 2%). These findings essentially reflect the characteristics of the current IBDV epidemic in China: the co-existence of multiple genotype strains; the long-term threat of the A3B3 genotype strain, which is characterized by high lethality; and the new threat posed by the A2dB1 genotype strain, which although does not kill the chickens directly, leading to serious immunosuppression.

Regarding the vvIBDV, previous epidemiological data have revealed that the highly lethal HLJ0504-like strain (A3B3 genotype) has, for long, been a major epidemic strain in China (21). Moreover, this strain has been reported worldwide in Pakistan (22), India (23), Bangladesh (24), Thailand (24), Vietnam (25), South Korea (13), Venezuela (26), and Nigeria (27). The findings of the present study have confirmed that the A3B3 genotype strain remains prevalent throughout China.

Since their emergence in the 1980s, varIBDVs have been continuously prevalent in North America, posing a considerable threat to the sustainability of the regional poultry industry. Conversely, in countries outside North America, the prevalence of the varIBDV strains had rarely been reported until recently. However, since 2017, nVarIBDV, which differs genetically from the varIBDVs prevalent in the United States, has been circulating in China (10, 11). Although this novel variant does not kill chickens, it can cause severe bursal atrophy and spleen swelling, resulting in pronounced immunosuppression (10, 11, 20). According to previous reports, nVarIBDV primarily infects commercial broilers aged between 3 and 6 weeks (10, 20). In the present study, we detected widespread prevalence of nVarIBDV in broilers, and also detected infection in layers and local breeds in China; this is of considerable significance for gaining a more comprehensive understanding of the epidemic status of nVarIBDV. However, whether nVarIBDV has the species tendency of susceptible chickens is not clear and needs further study. Notably, all chickens infected with nVarIBDV had previously been immunized, thereby indicating that nVarIBDV can, to a certain extent, evade the immuno-protection conferred by the existing vaccines. Compared to vvIBDV, the nVarIBDV is characterized by conspicuous antigenic differences. Consequently, some of the commercial vaccines targeting vvIBDV might not provide complete protection against nVarIBDV; this is deemed to be an important factor contributing to its widespread prevalence in immunized chickens. The key amino acids involved in the antigen differences need to be further identified.

Furthermore, we also found evidence indicating segment reassortment between the currently circulating strains. One incidence of segment reassortment occurred between two important circulating strains, namely A2dB3 (8.0%, 4/50), which is characterized by segment A from nVarIBDV and segment B from HLJ0504-like vvIBDV strains, such as IBDV-JS19-14601, IBDV-GD19-15010, IBDV-SD19-9804, and IBDV-SD19-9801. The second group showing reassortment comprises the A3B1 genotype viruses (6.0%, 3/50); in these, segments A and B are derived from HLJ0504-like vvIBDV and attenuated IBDV strains (such as IBDV-SD20-9102, IBDV-SD20-9103, and IBDV-SD20-9104), respectively. These observations accordingly highlight the complexity of the evolution and prevalence of IBDV, and consequently its prevention.

Segment reassortment is an important evolutionary genetic mechanism in IBDV, whose genome is composed of two double-stranded RNA segments. Random exchange between the genomes of IBDVs in different genotypes potentially leads to the generation of new IBDV genotypes (28). For example, in the present study, we detected a segment-reassortant A3B1 genotype strain, previously detected in China (29, 30), India (31), South Korea (32), Poland (33), Venezuela (26), Ethiopia (34), Nigeria (27), and Zambia (35); in this, segment A of the A3B2 genotype strain (vvIBDV) and segment B of the A8B1 genotype strain (attenuated strain) had undergone recombination. Furthermore, segment A of A8B1 IBDV and segment B of A3B2 IBDV recombined, resulting in A8B2 genotype strain (28, 36). A similar segment-reassortant stain of the A3BII genotype (containing segment A from vvIBDV and segment B from a serotype II strain) has been reported in recent years in the United States (37) and France (38, 39). In the present study, we found that 8% (4/50) of the detected strains were segment-reassortant A2dB3 genotype IBDV (segment A from the newly emerging A2dB1 genotype IBDV and segment B from the persistently circulating A3B3 genotype IBDV); its source and specific recombination mechanism require further study. It has long been considered that co-infection is a necessary prerequisite for the exchange of genome segments between the strains of different genotypes (28). Indeed, recent studies have shown that when IBDV strains of two genotypes co-infect the same cell, their respective viral replication factories co-accumulate, thereby providing an opportunity for segment reassortment among the different genotypes (40). Recent studies have shown that wild birds can act as carriers of IBDV to transmit the virus to chickens (41, 42), which might increase the risk of chickens infecting multiple genotypes of IBDV.

In conclusion, this study is the first to reveal the current novel epidemic characteristics of IBDV in major poultry-raising regions of China since the emergence of nVarIBDV. Our major findings comprise the identification of co-existence of strains of multiple genotypes and of the newly emerging nVarIBDV strain A2dB1 and the persistently circulating vvIBDV strain A3B3 as the two important epidemic strains. Furthermore, we found evidence indicating that segment reassortment between circulating strains has resulted in the generation of new genotypes. The complex nature of the current epidemic status of IBDV poses new challenges for comprehensive prevention.

## Data Availability Statement

The original contributions presented in the study are included in the article/Supplementary Material, further inquiries can be directed to the corresponding author/s.

## Author Contributions

XQ: conceptualization. NJ and XQ: methodology. NJ and YW: software. WZ, XN, MH, YG, AL, LG, KL, QP, CL, YZ, HC, and XW: validation. NJ, YW, and XQ: formal analysis. XQ and NJ: investigation. XQ: resources. XQ and NJ: data curation. NJ: writing—original draft preparation. XQ: writing—review and editing. NJ and XQ: visualization. XQ: supervision, project administration, and funding acquisition. All authors have read and agreed to the published version of the manuscript.

## Funding

This study was supported by the Heilongjiang Provincial Natural Science Foundation of China (Nos. ZD2020C006 and TD2019C003), National Natural Science Foundation of China (No. 32072852), the National Key Research and Development Program of China (Nos. 2016YFE0203200 and 2017YFD0500704), the Heilongjiang Province Foundation for the National Key Research and Development Program of China (GX18B011), China Agriculture Research System (CARS-41-G15).

## Conflict of Interest

The authors declare that the research was conducted in the absence of any commercial or financial relationships that could be construed as a potential conflict of interest.

## Publisher's Note

All claims expressed in this article are solely those of the authors and do not necessarily represent those of their affiliated organizations, or those of the publisher, the editors and the reviewers. Any product that may be evaluated in this article, or claim that may be made by its manufacturer, is not guaranteed or endorsed by the publisher.
