# Supplementary material for: Genotyping and Molecular Characterization of Infectious Bursal Disease Virus Identified in Important Poultry-Raising Areas of China During 2019 and 2020
Source: Front Vet Sci. 2021 Dec 1;8:759861. doi: 10.3389/fvets.2021.759861 (PMC8671459; doi:10.3389/fvets.2021.759861)
Supplement: Supplementary file 1 [file Table_1.DOC]

Supplementary Table 1

The HVR of segment A of reference IBDV strains.

| Genogroup | Phenotype^1)^ | IBDV strain | GenBank No. |
| --- | --- | --- | --- |
| A1 | C | IM USA | AY029166 |
|  |  | Lukert USA | AY918948 |
|  |  | 752 Morocco | MF142572 |
| A2 a^2)^ | Var | E Del USA | DD187400 |
|  |  | Variant E USA | AF133904 |
| b^2)^ |  | T1 USA | AF281238 |
|  |  | 9109 USA | AY462027 |
| c^2)^ |  | GLS USA | AY368653 |
|  |  | 7 Ohio USA | MF142583 |
| d^2)^ |  | SHG19 China | MH879092 |
|  |  | SHG120 China | MH879110 |
| A3 | VV | OKYM Japan | D49706 |
|  |  | HLJ0504 China | GQ451330 |
|  |  | Gx China | AY444873 |
|  |  | 02015.1 Venezuela | AJ878909 |
|  |  | HBL-07-15 India | KT630853 |
|  |  | 217/13 Poland | KX759610 |
|  |  | 276 Jordan | MF142517 |
|  |  | PK2 Pakistan | MF996499 |
| A4 | d | 741 UAE | MF142569 |
|  |  | MG4 Brazil | JN982252 |
| A5 | V/C Recomb | 760 Mexico | MF142575 |
|  |  | 94 Mexico | MF142589 |
| A6 | ITA | 772 KSA | MF142577 |
|  |  | 751 KSA | MF142571 |
|  |  | ITA-04 Italy | JN852988 |
| A7 | Australia | 06/95 Australia | AF148080 |
|  |  | 05-5 Australia | AF381011 |
|  |  | 429 Russia | MF142536 |
| A8 | AT | D78 USA | AF499929 |
|  |  | CEF94 Netherlands | AF133904 |
|  |  | NB China | AY319768 |
|  |  | CU-1 Germany | X16107 |
|  |  | Gt China | DQ403248 |
| BII | serotype II | OH Canada | U30818 |

^1)^ C, classic strain; Var, variant strain; vvIBDV, very virulent strain; dIBDV, distinct

strain; V/C Recomb, variant / classic recombinant strain; ITA, ITA-like strain; Australia, Australian strain; AT, attenuated strain; Serotype II, Serotype II IBDV. ^2)^ Lineages under genogroup A2.

Supplementary Table 2

The B-marker of Segment B of reference IBDV strains.

| Genogroup | Phenotype^1)^ | IBDV strain | GenBank No. |
| --- | --- | --- | --- |
| B1 | C | IM USA | AY029165 |
|  | Var | Variant E USA | AF133905 |
|  | AT | NB China | AY654284 |
|  |  | Gt China | DQ403249 |
|  | nVar | SHG19 China | MH879045 |
| B2 | vv | D6948 Netherlands | AF240687 |
|  |  | HK46 China | AF092944 |
|  |  | OKYM Japan | D49707 |
|  |  | UK661 France | NC-004179 |
| B3 | HLJ0504-like | HLJ0504 China | GQ451331 |
|  |  | Gx Chian | AY705393 |
|  |  | HBL-07-15 India | KT630845 |
|  |  | 02015.1 Venezuela | AJ880090 |
|  |  | PK2 Pakistan | MF996500 |
|  |  | SK53 Thailand | KJ198845 |
| B4 | Trans | Bug/03 Poland | KX759556 |
|  |  | li4129/2014 Poland | MG739299 |
|  |  | 217/13 Poland | KX759559 |
|  |  | 117/14 Poland | KX759562 |
| BII | Serotype II | OH Canada | U30819 |

^1)^C, classic strain; Var, variant strain; AT, attenuated strain; nVar, novel variant strain; VV, very virulent strain; HLJ0504-like, HLJ0504-like strain; Trans, transitional-lineage strain; Serotype II, serotype II IBDV.
